# Factors Influencing Childhood Immunization in Uganda

**DOI:** 10.3329/jhpn.v31i1.14756

**Published:** 2013-03

**Authors:** Edward Bbaale

**Affiliations:** Centre for Global Development, Washington, D.C. and Makerere University-Kampala, Uganda

**Keywords:** BCG vaccine, DPT vaccine, Polio vaccine, Full immunization, Measles vaccine, Uganda

## Abstract

This paper investigates the factors associated with childhood immunization in Uganda. We used nationally-representative data from Uganda Demographic and Health Survey (UDHS) of 2006. Both bivariate and multivariate approaches were employed in the analysis. The bivariate approach involved generating average percentages of children who were immunized, with analysis of pertinent background characteristics. The multivariate approach involved employing maximum likelihood probit technique and generating marginal effects to ascertain the probability of being immunized, given the same background characteristics. It revealed that slightly over 50% of children in Uganda were fully immunized. Additionally, 89%, 24%, 52%, and 64% received BCG, DPT, polio and measles vaccines respectively. Factors which have a significant association with childhood immunization are: maternal education (especially at post-secondary level), exposure to media, maternal healthcare utilization, maternal age, occupation type, immunization plan, and regional and local peculiarities. Children whose mothers had post-secondary education were twice as likely to be fully immunized compared to their counterparts whose mothers had only primary education (p<0.01). Thus, gender parity in education enhancement efforts is crucial. There is also a need to increase media penetration, maternal healthcare utilization, and to ensure parity across localities and regions.

## INTRODUCTION

Immunization is a proven tool for controlling and eliminating life-threatening infectious diseases and is estimated to avert 2 to 3 million deaths each year. It is one of the most cost-effective health investments, with proven strategies that make it accessible to even the most hard-to-reach and vulnerable populations (1). Universal immunization of children against eight vaccine-preventable diseases [tuberculosis, diphtheria, whooping cough (pertussis), tetanus, hepatitis B, respiratory diseases caused by *Haemophilus influenzae,* polio, and measles] is crucial to reducing infant and child mortality (2,3,4,5,6,7). Indeed, immunization is a major health intervention for child survival throughout the world (8). Consequently, childhood immunization remains a key channel for the attainment of the Millennium Development Goal 4 (MDG 4) of reducing child mortality by two-thirds within 2015.

Thus, it is no wonder that WHO launched the Expanded Programme on Immunization (EPI) in 1974, and many developing countries adopted it. Despite this effort, over 24,000 children die of vaccine-preventable diseases every day around the world. This is equivalent to 1 child dying every 3.6 seconds, 16-17 children dying every minute, and just about 9 million children dying every year. Of these deaths in 2008, a bigger proportion occurred in sub-Saharan Africa (4.4 million) and South Asia (2.8 million) compared to Latin America, the Caribbean (0.2 million), and industrialized countries (0.1 million). The Government of Uganda is committed to achieving the targets set out in the recently-launched National Development Plan (NDP, 2010-2015), many of which are in line with the MDGs to be achieved by 2015. In spite of these efforts, huge challenges still remain. The under-five and infant mortality rates are still high at 137 and 78 per 1,000 livebirths respectively (9). In addition, Uganda's total fertility rate (TFR) of about 7 children per woman is one of the highest in the world (2). It is noteworthy that any efforts to reduce the total fertility rate will yield very little, if not matched with tremendous reductions in the infant mortality rate.

Therefore, it is clear that, despite the universal childhood immunization programme, especially in developing countries, like Uganda, poor child health still persists. This has aroused a lot of interest among government policy-makers and other stakeholders (especially donors) in understanding the factors influencing the use of childhood immunization services. A key issue of interest is whether the children are fully immunized against all vaccine-preventable diseases and the set of associated factors that may need policy intervention. This paper contributes to the body of knowledge on the factors influencing the attainment of a full immunization package by providing answers to the following questions: (i) controlling for other socioeconomic characteristics, does the level of education acquired by a woman affect her decision in terms of seeking full immunization for her child?, (ii) if so, which level of a woman's schooling has a significant impact on this decision?, and (iii) do other socioeconomic and environmental factors matter?

There is a growing body of literature exposing the factors associated with childhood immunization. Different authors have put forward various factors believed to be associated with parental healthcare-seeking behaviour toward their children. The most frequently-cited factor influencing childhood immunization is maternal education. A noteworthy strand of literature exposes the pathways through which maternal education influences healthcare-seeking behaviour (10,11,12,13). It is argued that maternal education is accompanied with: changes in attitudes/beliefs and practices, autonomy and decision-making, control over resources, accessibility to a well-paying job and educated spouses, and control over their fertility behaviour, all of which enhance healthcare-seeking. Furthermore, a number of authors expose maternal education as an important factor influencing childhood immunization (3,4,8,10,14,15,16,17,18,19,20,21). Other important factors found in the literature are education of the spouse (3), household wealth status (3,5,16,22,23) and distance to healthcare facilities (4,5,24,25,26,27,28), along with locational and regional differences (3,4,14,19), age-cohort of the mother (6,8), the use of antenatal care (8,20,29), immunization plan (16), birth order (19), occupation of parents (3), and gender of the child (8). The literature survey informed us of our choice of the variables included in the analysis. In addition, it has exposed the fact that no nationally-representative study had so far been undertaken in Uganda. The study by Nankabirwa *et al.* (21) focused on eastern part of the country and children of less than 6 months who are not fully immunized. Therefore, our study, using nationally-representative data and focusing on fully-immunized children, represents a real scenario.

## MATERIALS AND METHODS

We obtained approval from Macro International to use the data from Uganda Demographic and Health Survey (UDHS) of 2006. It is a nationally-representative survey of 8,531 women aged 15-49 years and 2,503 men aged 15-54 years. The survey collected information on vaccination coverage for 25,020 children aged 0-36 months born in the five years preceding the survey. Of them, 16,131 (64.5%) were aged 0-11 months, 7,623 (30.5%) were aged 12-23 months, and 1,266 (5%) were aged 24-36 months. To analyze the probability of being fully immunized, we considered children of 12-36 months since they are believed to have completed the immunization cycle. To analyze the probability of receiving the individual vaccines, we considered even the younger children; hence, we used the entire sample from the age-cohort of 0-36 months. The UDHS report also considered this cohort during their descriptive analysis (2). Information on vaccination coverage was collected in two ways in the UDHS: from vaccination cards shown to the interviewer and from mothers’ verbal reports. If the cards were available, the interviewer copied the vaccination dates directly onto the questionnaire. Of 7,581 children, 626 (8%) did not have cards, 3,965 (52%) had cards and were seen by the interviewer, 2,122 (28%) had cards but were not seen by the interviewer, and 868 (12%) no longer had their cards (missing). When there was no vaccination card for the child or if a vaccine had not been recorded on the card as being given, the respondent was asked to recall the vaccines given to her child (2). Therefore, this was a crude immunization coverage which does not accurately show children properly immunized or actually protected against disease.

For the survey, three sets of questionnaire were used, namely; household questionnaire, women's questionnaire, and men's questionnaire. Sampling was done in two stages—in the first stage, 321 clusters were selected from among a list of clusters sampled in the 2005-2006 Uganda National Household Survey (UNHS) to generate matching samples that can allow for linking of the 2006 UDHS health indicators to poverty data from the 2005-2006 UNHS. Additional 17 clusters were selected from the 2002 Census from Karamoja to increase the sample-size to allow for reporting of the Karamoja-specific estimates in the UDHS. Finally, 30 internally-displaced camps (IDCs) were selected from a list of camps compiled by the United Nations Office for Coordination of Human Affairs, completing a total of 368 primary sampling units (2). In the second stage, households in each cluster were selected based on a complete listing of households as per UNHS listing. However, in addition to the UNHS-sampled households, another 20 households were randomly selected in each cluster (2). Uganda Bureau of Statistics (UBOS) recruited and trained staff to serve as supervisors, field editors, male and female interviewers, field coordinators, and health technicians. The UBOS, Macro International and invited experts from government ministries led the four-week training that included lectures, presentations, practical demonstrations, and practice interviewing in small groups as well as two days of field practice (2).

Our dependent variables were constructed as follows: (a) fully immunized: equals 1 if a child received the eight dozes against vaccine-preventable diseases and zero otherwise, (b) BCG: 1 if a child received BCG and zero otherwise, (c) DPT: 1 if a child received the three doses of DPT and zero otherwise; (d) Polio: 1 if a child received the three doses of polio vaccine and zero otherwise, and (e) Measles: 1 if a child was immunized against measles and zero otherwise.

We controlled for a number of independent variables guided by the previous literature. Maternal education was categorized into four outcomes as follows: 0=no education, 1=primary education, 2=secondary education, and 3=post-secondary education (tertiary and university). In all our regressions, no education was the reference category. Educational attainment of men was categorized in the same way. As shown in Table 1, age of the mother was broken into five-year age-cohorts as: 15-19, >19-24, >24-29, >29-34, >34-39, >39-44, >44-49. In all our regressions, 15-19 age-cohort was used as the reference category. Location was defined as equal to 1 if a mother was in the urban area and zero otherwise. Nine region dummies were defined as 1=Central 1, 2=Central 2, 3=Kampala, 4=East Central, 5=Eastern, 6=North, 7=West Nile, 8=Western, and 9=Southwest. The Central 1 was the reference category. We defined three religious dummies as 1=Catholics, 2=Protestants, 3=Muslims, and 4=Others. The category ‘Others’ included Evangelicals (born-again Christians), Seventh Day Adventists, Orthodox, and Traditionalists/Non-believers. Catholics were used as the reference category. The wealth index is provided in the dataset in terms of quintiles; 1=Poorest, 2=Poor, 3=Middle, 4=Rich, 5=Richest. The poorest quintile was used as the reference category. Occupation of the mother and spouse was categorized as follows: 1=White-collar job, 2=Services/sales, 3=Agriculture, 4=Blue-collar job. The white-collar workers were used as the reference category. Exposure to media was categorized as follows: 1=Not at all, 2=Less than once a week, 3=At least once a week, 4=Every day. Those who had no exposure to media at all were used as the reference category.

**T1able 1.** Distribution of 30,090 women aged 15-49 years, surveyed in the UDHS by key characteristics

Characteristics	Number	Percentage
Woman's education: No education (ref.)	9,470	32
Primary	17,463	58
Secondary	2,632	9
Post-secondary	525	2
Partner's education: No education (ref.)	3,892	14
Primary	17,648	62
Secondary	5,349	19
Post-secondary	1,695	6
Location: Urban (ref.)	3,166	11
Rural	26,924	90
Region: Central 1 (ref.)	2,670	9
Central 2	3,059	10
Kampala	1,506	5
East Central	3,657	12
Eastern	3,551	12
North	6,502	22
West Nile	2,411	8
Western	3,450	12
Southwest	3,284	11
Age-cohort: 15-19 years (ref.)	436	2
>19-24	2,790	9
>24-29	4,918	16
>29-34	6,296	21
>34-39	5,980	20
>39-44	5,027	17
>44-49	4,643	15
Wealth quintiles: Poorest (ref.)	7,378	25
Poor	6,090	20
Middle	5,882	20
Rich	5,722	19
Richest	5,018	17
Woman's occupation: White-collar job (ref.)	673	2
Services/sales	3,253	12
Agriculture	22,719	80
Blue-collar job	1,711	6
Partner's occupation: White-collar job (ref.)	2,430	8
Services/sales	3,750	13
Agriculture	18,638	63
Blue-collar job	4,642	16
Children's mmunization card: No card (ref.)	626	8
Card available and seen	3,965	52
Card not seen	2,122	28
No longer have the card (missing)	868	12

Source: author's computations from UDHS 2006

During the analysis, we employed both bivariate and multivariate approaches. The bivariate approach involved generating average percentages of children who were vaccinated, with background characteristics. Under the multivariate approach, maximum likelihood probit technique was used. After a probit estimation, we generated marginal effects to interpret the results as probabilities of being vaccinated, given the background characteristics.

## RESULTS

Results are presented from both bivariate and multivariate analyses. Our results are generated using sample weights and, hence, these are nationally representative. Table 2 shows the bivariate analysis. It reveals that, on average, 54% of children in Uganda were fully immunized, 89% received a full dose of BCG, 24% received DPT, 52% received polio, and 64% received the measles vaccine. The percentage of immunized children increased with maternal education; 63% of children whose mothers had post-secondary education were immunized compared to 53% of children having mothers with no education. Looking at the individual vaccines, the positive impact of maternal education is very clear. The findings of multivariate analysis in Table 3 confirm the bivariate results. Children whose mothers had at least primary education increased the probability of being fully immunized by 8-14% (p<0.05) compared to the counterparts with no education. Children whose mothers had at least secondary education were 6-7% (p<0.05) more likely to receive the three doses of DPT and polio vaccines compared to the counterparts having mothers with no education. Children whose mothers had at least primary education were 7-11% (p<0.01) more likely to be vaccinated against polio compared to the counterparts having mothers with no education.

Education of partner of the mother also portrayed the same picture as that of maternal education for full immunization and also for individual vaccines (Table 2). However, partner's education is statistically insignificant in the multivariate analysis and, hence, the coefficients have not been reported in Table 3. The wealth status of the household is marginally significant in the regression analysis for polio and measles vaccines. Children from the richest wealth quintile increased the probability of being vaccinated against polio and measles by 7% (p<0.1) compared to the counterparts in the poorest quintile (Table 3). Considering the bivariate findings, 53% and 67% of children in the richest quintile received polio and measles vaccines compared to 48% and 65% of the counterparts in the poorest quintile respectively.

The findings also reveal interesting locational and regional differences; 58% of children in urban areas were fully immunized compared to 53% of children in rural areas. A similar pattern is observed by looking into the individual vaccines (Table 2). Of the 9 regions, the North and West Nile had the highest percentage of children fully immunized and those who received BCG (59% and 93% respectively) while the lowest percentage was 40% and 75% for Central 1 respectively. The North had the highest percentage of children (32%) who received the three doses of DPT while the lowest percentage was 12% for Central 1. Western and Southwest had the highest percentage of children (58%) who received the three doses of polio while the lowest percentage was 44% for Central 1. The North had the highest percentage of children immunized against measles (71%) while the lowest percentage was 54% for Central 1 (Table 2). These differences were also confirmed in the multivariate analysis where we undertook joint significance tests for the regional dummies in our regressions. It revealed that our regional dummies are jointly significant in all our regressions (prob>chi^2^=0.0000 for all) (Table 3).

There are also differences owing to religious affiliation. Children from Muslim families reduced the probability of receiving the 3 doses of DPT by 3% (p<0.05) compared to the counterparts from Catholic families (Table 3). Children belonging to ‘Other’ religions increased the probability of being vaccinated against polio by 7-9% (p<0.05) compared to the counterparts belonging to Catholic religion (Table 3). Considering the bivariate analysis in Table 2, a high percentage of Catholic children were fully immunized (56%) compared to the rest, with the worst performer being Protestants (51%) (Table 2). A similar pattern was observed for individual vaccines apart from polio vaccine where children belonging to ‘Other’ religions had the highest percentage (58%) of children vaccinated against polio compared to the rest (Table 2).

The importance of prenatal care is reflected in our findings; 48% of children whose mothers sought professional prenatal care were fully immunized compared to 34% of children whose mothers never sought professional prenatal care (Table 2). Children whose mothers sought professional prenatal care increased the probability of being fully immunized, receiving DPT, polio vaccine, and measles vaccine by 9% (p<0.01), 3% (p<0.1), 6% (p<0.05), and 6% (p<0.05) respectively compared to the counterparts whose mothers never sought professional prenatal care (Table 3).

**T1able 2.** Average percentage of children who were fully immunized and received a full doze of each vaccine

Characteristics	No. of children	Fully-immunized (%)	BCG (%)	DPT (%)	Polio (%)	Measles (%)
Woman's education: No education (ref.)	1,824	53	88	28	48	64
Primary	4,686	53	88	22	52	63
Secondary	896	58	90	26	54	67
Post-secondary	185	63	97	30	63	71
Partner's Education: No education (ref.)	799	52	88	28	44	64
Primary	4,349	52	88	22	52	63
Secondary	1,527	56	90	27	53	66
Post-secondary	458	62	95	28	57	73
Location: Urban (ref.)	847	58	92	30	51	68
Rural	6,744	53	88	24	52	64
Region: Central 1 (ref.)	663	40	75	12	44	54
Central 2	645	51	83	20	55	62
Kampala	454	57	91	30	51	66
East Central	920	49	88	23	46	57
Eastern	971	50	90	24	53	61
North	1,647	59	93	32	50	71
West Nile	625	59	93	26	52	66
Western	862	58	92	24	58	69
Southwest	804	55	82	21	58	64
Religion: Catholic (ref.)	3,484	56	90	28	51	66
Protestant	2,500	51	88	21	53	64
Muslim	862	52	86	21	48	61
Other	486	54	88	24	58	61
Wealth quintiles: Poorest (ref.)	1,906	55	91	29	48	65
Poor	1,645	53	88	24	51	63
Middle	1,423	52	86	21	54	61
Rich	1,355	52	87	20	54	64
Richest	1,262	57	90	28	53	67
Prenatal care: Yes (ref.)	4,253	48	88	19	50	56
No	467	34	78	14	38	45
Exposure to media: not at all (TV) (ref.)	6,825	53	88	24	51	64
Less than once a week	285	58	88	31	54	66
At least once a week	207	59	88	25	54	68
Every day	268	61	93	35	52	69
Age-cohort: 15-19 years (ref.)	397	36	88	14	43	43
>19-24	1,918	52	88	23	50	63
>24-29	2,027	55	89	26	51	65
>29-34	1,627	55	88	24	54	65
>34-39	1,011	56	87	26	54	67
>39-44	467	59	90	29	55	70
>44-49	144	64	90	31	53	78
Child's gender: Male (ref.)	3,713	53	88	24	51	63
Female	3,878	54	89	25	52	65
Woman's occupation: White collar job (ref.)	183	66	96	34	62	74
Services/sales	785	57	89	31	48	69
Agriculture	5,623	54	89	24	53	64
Blue-collar job	396	53	87	25	50	66
Partner's occupation: White-collar job (ref.)	531	63	92	30	54	74
Services/sales	950	55	87	23	53	64
Agriculture	4,455	53	88	24	52	63
Blue-collar job	1,342	52	89	23	50	64
Immunization card: No card (ref.)	626	4	10	3	3	9
Had card and seen	3,965	59	98	8	73	64
Had card but not seen	2,120	56	92	49	35	75
No longer had card (missing)	868	61	95	56	31	78
Total	7,591	54	89	24	52	64

Source: Author's own calculations from UDHS 2006

Exposure to the media is also an important factor in our findings; 61% of children whose mothers watched TV daily were fully immunized compared to 53% of their counterparts who had no access at all. A similar pattern is found while considering the percentage of children who received a full dose of the individual vaccines. Children whose parents watched TV at least once a week increased the probability of being fully immunized by 9-15% (p<0.05) compared to the counterparts whose parents had no access to media at all. Children whose parents watched TV less than once a week and daily increased the probability of receiving DPT by 9-10% (p<0.01) compared to the counterparts whose parents had no access to media at all.

Whereas there are minor differences across age-groups of mothers, the percentage of children fully immunized in totality and along the individual vaccines increased with the mother's age; 64% of children whose mothers were in 45-49 years age-cohort were fully immunized compared to 36% of children whose mothers were in the 15-19 years age-cohort (Table 2). A similar pattern was observed for the full dose of the individual vaccines. Children whose mothers were in 20-49 years age-cohort increased the probability of being fully immunized, receiving DPT, polio and measles vaccine by 9-30% (p<0.01), 5-23% (p<0.05), 9-23% (p<0.01), and 13-35% (p<0.01) respectively (Table 3).

Our findings reveal the importance of the type of occupation of parents of the child. Children whose mothers were in agriculture and blue-collar jobs reduced the probability of receiving the 3 doses of DPT by 8% (p<0.05) and 5% (p<0.1) respectively compared to the counterparts whose mothers had white-collar jobs. Similarly, children whose fathers were in agriculture and blue-collar jobs reduced the probability of being fully immunized and immunized against measles by 8-10% (p<0.01) and 11-15% (p<0.01) respectively compared to the counterparts whose fathers had white-collar jobs (Table 3). Findings in the bivariate analysis in Table 2 confirmed these regression results; 66% of children whose mothers had a white-collar job were fully immunized compared to 53%, 54%, and 57% of children whose mothers were in blue-collar jobs, agriculture, and services respectively. A similar pattern was observed for partner's occupation and for individual vaccines.

Having an immunization card is shown to be important for full immunization. Children having immunization cards that were seen by the interviewer increased the probability of being fully immunized, receiving BCG, DPT, polio vaccine, and measles vaccine by 67% (p<0.01), 72% (p<0.01), 8% (p<0.01), 76% (p<0.01), and 64% (p<0.01) respectively compared to the counterparts who had no card at all. Children having immunization card but not seen by the interviewer increased the probability of being fully immunized, receiving BCG, DPT, polio, and measles vaccine by 66% (p<0.01), 21% (p<0.01), 58% (p<0.01), 52% (p<0.01), and 64% (p<0.01) respectively compared to the counterparts who had no card at all. Children who no longer had their cards increased the probability of being fully immunized, receiving BCG, DPT, polio, and measles vaccine by 60% (p<0.01), 10% (p<0.01), 67% (p<0.01), 46% (p<0.01), and 53% (p<0.01) respectively compared to the counterparts who had no card at all (Table 3). It might be the case that some health centres, especially those in the rural areas, never issued immunization cards to children mainly due to shortage of supply. Findings in the bivariate analysis presented in Table 2 confirmed the regression findings. Only 4% of children who had no immunization card were fully immunized compared to 59%, 56%, and 61% of children who had a card that was seen, a card which was not seen, and no longer had their cards respectively. However, the empirical and descriptive findings attached to children who had no immunization cards suffered a setback of a recall bias and, therefore, should be interpreted and considered for policy with caution.

## DISCUSSION

This paper investigated the factors associated with childhood immunization status in Uganda. The finding that slightly over 50% of children were fully immunized is worrying in the face of very high infant and child mortality. This may probably be due to the ignorance of mothers or parents concerning the right dosage that a child is required to get. This is especially true for vaccines, like DPT and polio, that require three independent doses where some children may receive one dose or up to the second dose and do not complete the full course. Government effort is urgently needed to increase coverage of immunization by educating the population on the right doses of each vaccine and importance of completing it. There is a discrepancy between the level of vaccination coverage between DPT (24%) and polio vaccine (52%). These vaccines are often administered at the same time and, hence, their coverage rates are expected to be similar. According to the UDHS report, differences in coverage between DPT and polio result, in part, from stock-outs of vaccines (2).

Maternal education has been highlighted as an important predictor of full childhood immunization and receiving individual vaccines. Literature attributes this to changes that accompany maternal education, such as changes in attitudes, traditions and beliefs, increased autonomy and control over household resources, which enhance healthcare-seeking (10,11,12,13). Our findings are in line with those from previous studies that articulated the importance of maternal education in the demand for childhood immunization (3,4,8,10,14,15,16,17,18, 19,20,21). Thus, the Universal Secondary Education Programme of the Government of Uganda is a commendable start that needs to be propelled to higher levels, with gender parity at the forefront.

We also found significant regional differences in the immunization coverage in Uganda. This may probably be attributed to the long distance of some regions from the vaccine distribution centres and from health facilities. Previous authors also found regional and locational disparities to be important (3,4,14,19). Therefore, there is a need for the government effort to reach the disadvantaged regions and locations. The Government of Uganda should target rural areas by stocking the required vaccines in the community health centres or clinics. In addition, sensitization campaigns, especially targeting the uneducated, may go a long way in achieving this objective. In many of the cases, rural areas are disadvantaged due to poor road networks, especially during rainy seasons. Improving the transport facilities may also help solve the problem.

Mothers who sought prenatal care were associated with a higher likelihood of their children being fully immunized compared to the counterparts who did not seek prenatal care. This can be attributed to learning sessions that mothers are exposed to during prenatal care where the importance of timely immunization of the baby is emphasized. Our results are in agreement with the previous literature concerning the importance of prenatal care utilization (8,20,29). Women should be sensitized to seek maternal healthcare services during pregnancy and childbirth as a way of having a continuous relationship with health workers even after childbirth.

Exposure to the media is significantly associated with childhood immunization. This can be attributed to the sensitization messages that parents receive through media to get their children immunized. The Government and other stakeholders should continuously advertise through media the importance of childhood immunization. In addition, government policy should ensure that various media channels fully penetrate the population as an effective way to disseminate the information.

We also found that the likelihood of childhood immunization increases with maternal age. This may be attributed to experience accumulated over time on the importance of immunization and also on the fatalities that have occurred to children due to lack of immunization. This finding is in line with that in the previous literature (6,8). There is a need for more sensitization among young mothers on the importance of childhood immunization.

Mother's occupation and that of her partner are important in the attainment of full childhood immunization. Children whose parents held white-collar jobs were more advantaged compared to those in agriculture, blue-collar jobs, and services/sales. This may be attributed to the ease of accessibility to information that white-collar workers may have. It may also be due to accessibility to health facilities since the majority of white-collar workers are located in urban areas. Previous literature also found the type of occupation to be important in influencing childhood immunization (3). Government policy should be designed to target the disadvantaged, especially those in the agricultural sector by establishing village-level clinics with full stock of vaccines. It is not surprising that wealth status was not significant in influencing full childhood immunization since immunization is universal in Uganda. However, literature on other countries exposes the importance of the wealth status of the household (3,5,16,22,23).

Having an immunization plan in the form of an immunization card was decisively important in our analysis. Mothers having a child immunization card can easily follow the immunization schedule and be able to attain timely immunization for their children. Previous researchers did find the importance of having an immunization plan (16). Having a well-designed immunization card, with a clearly-labelled schedule, can go a long way in enhancing childhood immunization by reducing the forgetfulness of parents.

**T1able 3.** Probability of a child being fully immunized in totality and by individual vaccine (marginal effects after a probit estimation)

Variable	Fully immunized		BCG		DPT		Polio		Measles	
Woman's education: Primary	0.0755***	-	0.00210	-	0.0169	-	0.0350	-	0.0738***	-
	(0.000241)	-	(0.852)	-	(0.175)	-	(0.105)	-	(0.000476)	-
Secondary	0.137***	-	0.0104	-	0.0561***	-	0.0753**	-	0.112***	-
	(1.74e-05)	-	(0.536)	-	(0.00879)	-	(0.0233)	-	(0.000467)	-
Post-secondary	0.121**	-	0.0240	-	0.0658*	-	0.0624	-	0.0983*	-
	(0.0205)	-	(0.415)	-	(0.0692)	-	(0.254)	-	(0.0642)	-
Wealth status: poor	0.00121	0.0158	-0.00987	0.00195	-0.00198	0.00316	-0.0219	-0.0147	0.0172	0.0358
	(0.961)	(0.530)	(0.491)	(0.887)	(0.895)	(0.840)	(0.389)	(0.573)	(0.493)	(0.162)
Middle	0.000371	0.00922	-0.0171	-0.0105	-0.00601	-0.0110	0.0123	0.0165	0.0225	0.0313
	(0.989)	(0.750)	(0.298)	(0.512)	(0.725)	(0.535)	(0.673)	(0.586)	(0.428)	(0.288)
Rich	0.0176	0.0293	0.00456	0.0125	-0.0233	-0.0289	0.00646	0.0207	0.0370	0.0430
	(0.545)	(0.341)	(0.779)	(0.431)	(0.180)	(0.114)	(0.832)	(0.520)	(0.213)	(0.168)
Richest	0.0261	0.0384	0.00252	-0.000287	-0.00182	-0.00559	0.0185	0.0684*	0.0717**	0.0575
	(0.462)	(0.328)	(0.897)	(0.989)	(0.933)	(0.814)	(0.618)	(0.0950)	(0.0469)	(0.149)
Location: Rural	-0.0399	0.0119	-0.0171	-0.0118	-0.0293	0.00791	-0.0105	0.0121	-0.0506	-0.0170
	(0.259)	(0.779)	(0.381)	(0.607)	(0.182)	(0.752)	(0.775)	(0.783)	(0.168)	(0.702)
Region: Central 2	0.0604	0.0447	0.0129	0.0182	0.120***	0.143***	0.0730*	0.0857*	0.0211	0.000999
	(0.124)	(0.314)	(0.435)	(0.293)	(0.000464)	(0.000513)	(0.0721)	(0.0629)	(0.592)	(0.982)
Kampala	0.0106	0.00192	0.0321	0.0469*	0.0783**	0.131**	-0.0184	-0.00554	-0.0572	-0.0856
	(0.834)	(0.976)	(0.169)	(0.0549)	(0.0358)	(0.0126)	(0.725)	(0.933)	(0.274)	(0.194)
East Central	0.0327	-0.00791	0.0424***	0.0429***	0.179***	0.201***	-0.0533	-0.0816*	-0.0557	-0.0910**
	(0.375)	(0.847)	(0.00235)	(0.00298)	(1.98e-07)	(7.14e-07)	(0.159)	(0.0534)	(0.133)	(0.0288)
Eastern	0.0766**	0.0421	0.0523***	0.0516***	0.189***	0.215***	0.0604	0.0416	0.0230	-0.0144
	(0.0418)	(0.309)	(0.000137)	(0.000237)	(8.52e-08)	(1.66e-07)	(0.122)	(0.334)	(0.543)	(0.730)
North	0.153***	0.112***	0.0443***	0.0488***	0.190***	0.207***	0.0550	0.0297	0.112***	0.0736*
	(4.29e-05)	(0.00681)	(0.00368)	(0.00193)	(9.43e-09)	(4.48e-08)	(0.155)	(0.487)	(0.00275)	(0.0760)
West Nile	0.121***	0.0685	0.0546***	0.0536***	0.222***	0.224***	0.0190	-0.00750	0.0531	-0.00734
	(0.00268)	(0.120)	(0.000229)	(0.000412)	(2.64e-08)	(5.20e-07)	(0.648)	(0.869)	(0.190)	(0.869)
Western	0.166***	0.123***	0.0546***	0.0522***	0.159***	0.174***	0.147***	0.118***	0.102***	0.0613
	(1.25e-05)	(0.00312)	(2.87e-05)	(0.000125)	(4.53e-06)	(1.38e-05)	(0.000176)	(0.00626)	(0.00713)	(0.143)
Southwest	0.146***	0.113***	0.0241	0.0246	0.167***	0.177***	0.135***	0.126***	0.111***	0.0762*
	(0.000148)	(0.00747)	(0.116)	(0.124)	(2.49e-06)	(1.42e-05)	(0.000677)	(0.00400)	(0.00360)	(0.0720)
Joint significance test for regional dummies: chi^2^ (8)	38.80	26.24	32.72	28.78	41.51	34.74	48.37	46.03	45.27	37.92
Prob>chi^2^	(0.0000)	(0.0000)	(0.0001)	(0.0003)	(0.0000)	(0.0000)	(0.0000)	(0.0000)	(0.0000)	(0.0000)
Religion: Protestant	-0.0191	-0.0170	0.00917	0.0112	-0.0152	-0.0157	0.00281	0.00485	0.0137	0.0182
	(0.292)	(0.383)	(0.359)	(0.279)	(0.170)	(0.189)	(0.883)	(0.812)	(0.461)	(0.360)
Muslim	-0.00182	0.00104	0.00768	0.00970	-0.0315**	-0.0350**	-0.00441	0.0132	0.0106	0.0206
	(0.946)	(0.972)	(0.597)	(0.528)	(0.0436)	(0.0436)	(0.876)	(0.674)	(0.702)	(0.501)
Other religion	-0.00847	0.00596	-0.00743	0.00260	-0.0162	-0.0144	0.0721**	0.0871**	-0.0314	-0.0204
	(0.791)	(0.865)	(0.677)	(0.888)	(0.402)	(0.500)	(0.0330)	(0.0180)	(0.339)	(0.567)
Exposure to media: <Once a week (TV)	0.0868**	0.0884*	0.000487	0.00800	0.0854***	0.0951***	0.0587	0.0462	0.0498	0.0424
	(0.0351)	(0.0564)	(0.982)	(0.735)	(0.00286)	(0.00301)	(0.167)	(0.335)	(0.236)	(0.375)
At least once a week	0.102**	0.152**	0.000370	-0.0317	0.0342	0.0534	0.0732	0.109*	0.0655	0.0886
	(0.0352)	(0.0121)	(0.988)	(0.336)	(0.278)	(0.177)	(0.145)	(0.0830)	(0.183)	(0.149)
Every day	0.0606	0.0172	0.0304	0.0268	0.0961***	0.0489	0.0406	-0.0317	0.0192	-0.00621
	(0.178)	(0.761)	(0.162)	(0.345)	(0.00246)	(0.186)	(0.387)	(0.590)	(0.677)	(0.915)
Age cohort: 20-24 years	0.0922***	0.0636	-0.0274	-0.0233	0.0378	0.0357	0.0679**	0.0315	0.133***	0.103***
	(0.00532)	(0.114)	(0.175)	(0.328)	(0.101)	(0.215)	(0.0455)	(0.447)	(4.62e-05)	(0.00923)
>24-29	0.145***	0.104***	-0.00773	0.00168	0.0461**	0.0400	0.0890***	0.0370	0.167***	0.125***
	(1.27e-05)	(0.00931)	(0.692)	(0.940)	(0.0495)	(0.163)	(0.00920)	(0.369)	(3.17e-07)	(0.00144)
>29-34	0.145***	0.101**	-0.0144	-0.00607	0.0441*	0.0396	0.102***	0.0501	0.195***	0.153***
	(2.14e-05)	(0.0130)	(0.479)	(0.792)	(0.0688)	(0.177)	(0.00349)	(0.232)	(4.26e-09)	(0.000111)
>34-39	0.203***	0.152***	-0.00127	0.00407	0.0698**	0.0528*	0.159***	0.0982**	0.244***	0.199***
	(3.26e-08)	(0.000383)	(0.952)	(0.861)	(0.0104)	(0.0946)	(2.50e-05)	(0.0257)	(0)	(1.13e-06)
>34-39	0.247***	0.194***	0.0165	0.0263	0.127***	0.120***	0.186***	0.121**	0.295***	0.249***
	(3.57e-09)	(4.15e-05)	(0.465)	(0.253)	(0.000152)	(0.00188)	(1.55e-05)	(0.0132)	(0)	(1.96e-08)
>44-49	0.306***	0.244***	-0.0226	-0.0177	0.101**	0.0869*	0.230***	0.167***	0.346***	0.304***
	(6.89e-08)	(6.54e-05)	(0.506)	(0.611)	(0.0171)	(0.0555)	(8.82e-05)	(0.00807)	(0)	(9.70e-08)
Mother's occupation: Services/sales	-	0.0425	-	0.000364	-	-0.0103	-	-0.0113	-	0.0635
	-	(0.463)	-	(0.992)	-	(0.744)	-	(0.852)	-	(0.292)
Agriculture	-	-0.0351	-	-0.0294	-	-0.0808**	-	0.00384	-	-0.0415
	-	(0.532)	-	(0.350)	-	(0.0266)	-	(0.948)	-	(0.480)
Blue-collar job	-	-0.0410	-	-0.0647	-	-0.0499*	-	0.0154	-	-0.00433
	-	(0.515)	-	(0.206)	-	(0.0975)	-	(0.817)	-	(0.948)
Partner's occupation: Services/sales	-	-0.0541	-	0.00589	-	-0.00859	-	0.00552	-	-0.0776*
	-	(0.165)	-	(0.782)	-	(0.702)	-	(0.894)	-	(0.0638)
Agriculture	-	-0.0813**	-	0.00754	-	-0.0194	-	-0.0188	-	-0.114***
	-	(0.0210)	-	(0.701)	-	(0.350)	-	(0.611)	-	(0.00204)
Blue-collar job	-	-0.104***	-	-0.0190	-	-0.0217	-	-0.0490	-	-0.149***
	-	(0.00489)	-	(0.390)	-	(0.300)	-	(0.212)	-	(0.000157)
Prenatal care	0.0941***	0.0928***	0.00259	-0.00279	0.0280*	0.0215	0.0558**	0.0470	0.0630**	0.0584**
	(0.000435)	(0.00125)	(0.853)	(0.844)	(0.0710)	(0.206)	(0.0468)	(0.119)	(0.0229)	(0.0490)
Immunization card: Yes and seen	0.665***	0.661***	0.715***	0.714***	0.0745**	0.0692**	0.759***	0.754***	0.640***	0.640***
	(0)	(0)	(0)	(0)	(0.0126)	(0.0252)	(0)	(0)	(0)	(0)
Yes but not seen	0.663***	0.647***	0.208***	0.198***	0.578***	0.566***	0.521***	0.503***	0.637***	0.621***
	(0)	(0)	(0)	(0)	(0)	(0)	(0)	(0)	(0)	(0)
No longer had card (missing)	0.602***	0.595***	0.0989***	0.0967***	0.684***	0.677***	0.464***	0.455***	0.527***	0.521***
	(0)	(0)	(0)	(0)	(0)	(0)	(0)	(0)	(0)	(0)
Observations	4,546	3,962	4,539	3,957	4,537	3,956	4,541	3,959	4,532	3,950

p values in parentheses

*** p<0.01

** p<0.05

* p<0.1

The greatest strength of our study is its use of data from a nationally-representative survey, which enhances generalization of results for the entire country. However, the primary source of limitation is the recall bias. Data were collected retrospectively for the past five years and, hence, mothers may not be in a position to recall very well all the events that took place during childhood immunization, especially where the card was missing. However, this bias did not occur in the case where the card was seen or available, and, luckily enough, this constituted the majority in our analysis.

### Conclusions

This paper has provided insights into the factors associated with full childhood immunization in Uganda. This is expected to be of great importance to the policy-makers as the country trails with low childhood immunization, despite the universal immunization programme. Efforts are needed to enhance education, with gender parity at the forefront. There is also a need to strengthen the village outreach programme by establishing village-level clinics that are well-stocked with all vaccines to overcome regional disparities. Media outreach should also be increased among the population, and the Government can use this channel to disseminate a standard piece of information concerning the importance and doses of childhood immunization. Maternal healthcare services, such as prenatal care, should be enhanced so that there is a close contact between mothers, children, and health workers. Efforts are needed to create a comprehensive immunization plan labelled on an immunization card in a way that can easily be understood by the less educated. The future studies should find out which doses for an individual vaccine, say DPT, children require most―the first, second, or the third dose. This may help in the better understanding of the factors influencing full immunization.

## ACKNOWLEDGEMENTS

The author acknowledges the Centre for Global Development (CGD) for offering a Visiting Fellowship that provided the time to write this article. Thanks are also due to IDRC for sponsoring the Fellowship and to Makerere University for granting leave. However, the views expressed in this article are those of the author and not of CGD.
